# Associations between safety, tolerability, and toxicity and the reporting of health‐related quality of life in phase III randomized trials in common solid tumors

**DOI:** 10.1002/cam4.3390

**Published:** 2020-09-04

**Authors:** Ramy R. Saleh, Nicholas Meti, Domen Ribnikar, Hadar Goldvaser, Alberto Ocana, Arnoud J. Templeton, Bostjan Seruga, Eitan Amir

**Affiliations:** ^1^ Division of Medical Oncology & Hematology Department of Medicine Princess Margaret Cancer Centre University of Toronto Toronto ON Canada; ^2^ Department of Medical Oncology Institute of Oncology Ljubljana Ljubljana Slovenia; ^3^ Davidoff Cancer Center Beilinson Hospital Rabin Medical Center Petah Tikva Israel; ^4^ Sackler Faculty of Medicine Tel Aviv University Tel Aviv Israel; ^5^ Experimental Therapeutics Unit Medical Oncology Department Hospital Clínico San Carlos, and IdISSC Madrid Spain; ^6^ Centro de Investigación Biomédica en Red Cáncer (CIBERONC) Madrid Spain; ^7^ Centro Regional de Investigaciones Biomédicas Castilla‐La Mancha University Ciudad Real Spain; ^8^ Department of Oncology St. Claraspital Basel Switzerland; ^9^ Faculty of Medicine University of Basel Basel Switzerland

**Keywords:** oncology, quality of life, randomized controlled trials, treatment toxicity

## Abstract

**Background:**

Anti‐cancer drugs are approved typically on the basis of efficacy and safety as evaluated in phase III randomized trials (RCTs). Health‐related quality of life (HRQoL) is a direct measure of patient benefit, but is under‐reported. Here we explore associations with reporting of HRQoL data in phase III RCTs in common solid tumors.

**Methods:**

We searched ClinicalTrials.gov to identify phase III RCTs evaluating new drugs in adults with advanced cancers that completed accrual between January 2005 and October 2016. Data on HRQoL, safety, and tolerability comprising treatment‐related death, treatment discontinuation and commonly reported grade 3 or 4 adverse events (AEs) were extracted. Associations between these measures and reporting of HRQoL data were explored using logistic regression.

**Results:**

Of 377 phase III RCTs identified initially, 143 studies were analysed and comprised 55% positive trials and 90% industry sponsored trials. HRQoL was listed as an endpoint in 59% trials; and of these, only 65% reported HRQoL data. There were higher odds of reporting HRQoL data for positive trials (OR 2.05, *P* = .04) and trials published in journals with higher impact factor (OR 1.35, *P* = .01). Reporting of HRQoL was not associated with treatment‐related death (OR 1.25, *P* = .40) or treatment discontinuation (OR 1.12, *P* = .61), but was positively associated with dyspnea and dermatological adverse events.

**Conclusions:**

HRQoL is reported in only two‐thirds of RCTs that describe collecting such data. Reporting of HRQoL is associated with positive trial outcome and higher journal impact factor, but not associated with overall safety and tolerability of anti‐cancer drugs.

## Graphical abstract

HRQoL data remain underreported in phase III, cancer clinical trials that report the collection of such data. Herein, we demonstrate that reporting of HRQoL is associated with positive trial outcome and higher journal impact factor, but not with overall safety and tolerability of anti‐cancer drugs.

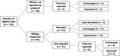

## 1 INTRODUCTION

A key objective of any medical therapy is to improve the duration or quality of survival. As such, overall survival and quality of life are definitive outcome measures in clinical trials. Intermediate efficacy endpoints are utilized commonly in trials of cancer therapy despite only few having been validated as surrogates for definitive outcomes. Quality of life is a direct measure of patient benefit,^1^ especially in patients receiving treatment with palliative intent. Quality of life data can also be used to support registration of new cancer drugs.^2^

The use of patient reported outcomes (PROs) has been used increasingly to evaluate the benefits of novel treatment.^3^, ^4^, ^5^ Health‐related quality of life (HRQoL) is a type of PRO measurement that refers to the multi‐dimensional assessment that includes features such as physical, psychological, social, and cognitive functioning.^6^ HRQoL is assessed using validated questionnaires to ensure the questions are standardized across all trial participants and also to attribute any differences between patient responses to true differences in perceptions of their outcomes as opposed to methodological differences.^7^ Importantly, HRQoL provides clinically meaningful information about how experimental anti‐cancer drugs and regimens impact the patients’ overall health, possibly reflecting the balance between treatment efficacy and toxicity.^8^

Despite its recognized value and recommendations to include HRQoL data in cancer clinical trials from multiple professional societies, including the American Society of Clinical Oncology (ASCO) and European Society for Medical Oncology (ESMO),^3^, ^9^, ^10^ these data remain underreported.^11^, ^12^, ^13^, ^14^ The reason for this is unclear. Therefore, here, we investigate whether safety and tolerability outcomes influence HRQoL data reporting in trials of common advanced cancer. We hypothesized that trials of more toxic experimental drugs will be associated with a lower odds of reporting HRQoL data.

## 2 METHODS

### 2.1 Identification of studies

We searched the ClinicalTrials.gov database to identify phase III randomized trials (RCTs) evaluating new drugs in adults with advanced breast, colorectal, lung, or prostate cancers. We included all completed or active trials that finished accrual between 1 January 2005 and 31 October 2016. This allowed for a minimum of 3 years of post‐accural follow‐up for reporting of trial results. We excluded trials evaluating supportive care agents, studies with different scheduling and/or dosing of the same agent, single‐arm studies, and trials not evaluating systemic therapy (such as trials exploring radiation, surgery, imaging [including screening], and chemoprevention). We also excluded trials that comprised exclusively of biomarker, pharmacokinetic, or pharmacodynamics analysis. We then searched for reporting of completed trials in the scientific literature. For some trials, a direct link to the full publication was available and the date of presentation at a scientific meeting was mentioned in the publication itself. The remaining trials were identified through a search of (MEDLINE [host: PubMed] and a supplementary search using Google Scholar) using the name of the trial and of the experimental drug. To complete the search, we reviewed the proceedings of the American Society of Clinical Oncology, European Society for Medical Oncology, and San Antonio Breast Cancer Symposium for follow‐up presentations and publications that reported HRQoL data. If no HRQoL reports were identified, we contacted the corresponding authors of the reports of efficacy data to request details on presentation dates and results.

### 2.2 Data extraction and synthesis

Two authors (DR and RR) extracted the following data from each of the identified RCTs: ClinicalTrials.gov identifier (NCT number), evaluated drug, type of experimental therapy (chemotherapy, endocrine therapy, targeted therapy, immunotherapy, other), cancer site, intent of the treatment (adjuvant/curative or palliative), date of reporting at a scientific conference, date of scientific publication (online publication, if applicable), trial result (positive or negative as reported in individual trials for the primary endpoint), primary endpoint and its result (eg hazard ratio), year of commencement of accrual, sponsor (industry vs non‐industry), the name of the study sponsor, journal impact factor (for studies published in full), reporting of a HRQoL endpoint, tool used for HRQoL assessment and where HRQoL was reported. We then collected the number of trial participants in both the experimental and control arms with each of the following safety and tolerability measures: treatment‐related death, treatment discontinuation without progression as well as the following individual adverse events (AEs): anemia, neutropenia, thrombocytopenia, diarrhea, stomatitis, hypertension, cardiotoxicity, fatigue, dermatological, dyspnea, and neuropathy.

### 2.3 Data synthesis and statistical analysis

Data were presented as means or proportions as appropriate. First we calculated the odds ratio (OR) for each safety and tolerability measure comparing experimental to control therapy in each trial. Calculation of OR differed based on the type of safety and tolerability measure. For treatment‐related death where absolute event rates were less than 1%, the Peto one‐step odds ratio method was utilized.^15^, ^16^ For treatment discontinuation where there were low absolute event rates and substantial variability in relative effect‐sizes, the Mantel‐Haenszel odds ratio method was used.^17^ Finally, for AEs, the DerSimonian and Laird random‐effects method was utilized and studies were weighted using the generic inverse variance approach.^18^ Associations between log‐transformed ORs for safety and tolerability measures and the reporting of HRQoL data were performed using univariable logistic regression analysis and were reported as OR together with their respective 95% confidence intervals (CI). Trends for reporting HRQoL data over time were explored using linear regression. Analyses were conducted using SPSS version 26 (IBM Corp, Armonk, NY). All statistical tests were two‐sided and statistical significance was defined as *P* < .05. No corrections were made for multiple significance testing.

## 3 RESULTS

### 3.1 Clinical trial characteristics

**FIGURE 1:**
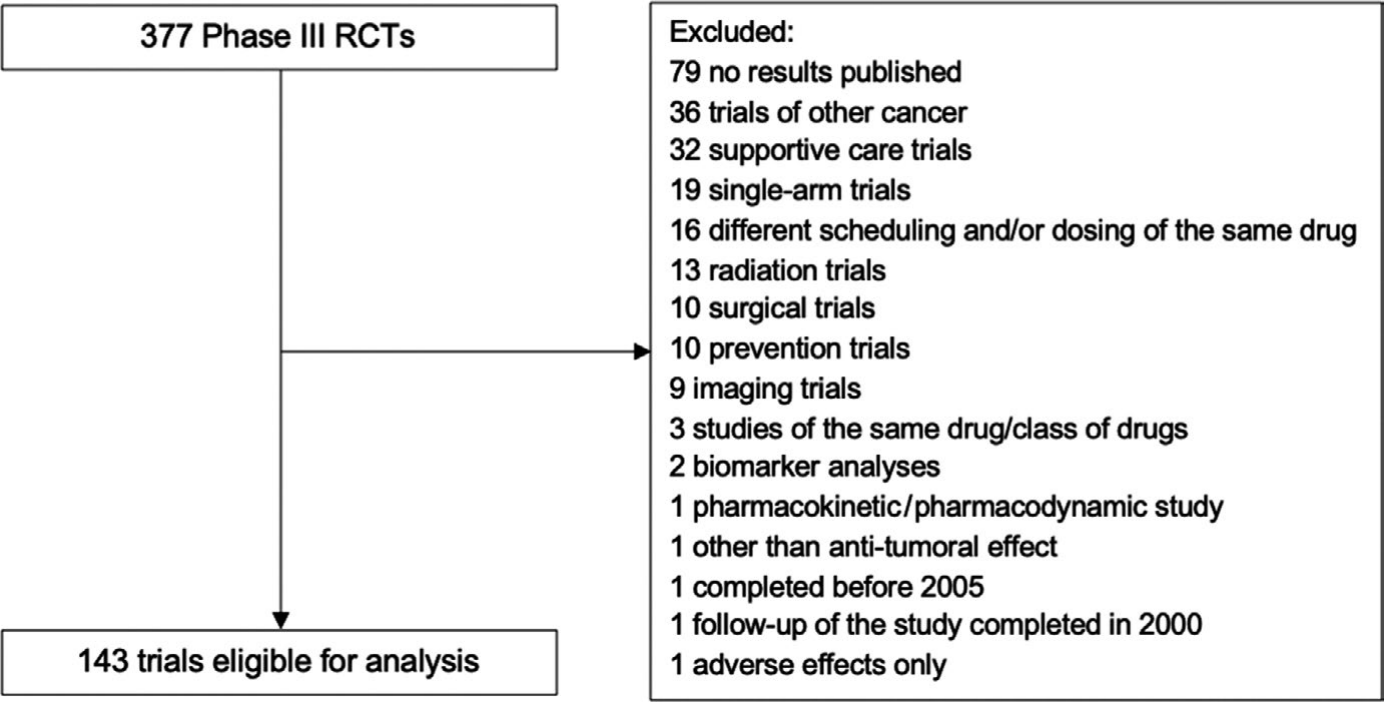
Flowchart of study selection. HRQoL, health‐related quality of life; RCT, Randomized controlled trial

A total of 377 phase III RCTs were identified initially and after exclusion of trials not meeting the selection criteria, a total of 143 studies were eligible for the analysis (see Figure 1 for study selection schema). The characteristics of included studies as well as the PRO measurement tools used in individual studies are shown in Table 1.

**TABLE 1:** Characteristics of the included trials

Characteristic	
HRQoL mentioned as endpoint	n = 143
Yes	84 (59%)
No	59 (41%)
HRQoL reported	
Initial trial reporting	47 (33%)
Post‐hoc reporting	12 (7%)
Not reported	84 (59%)
Trial outcome	
Positive	79 (55%)
Negative	60 (42%)
Did not report	4 (3%)
Cancer site	
Breast	40 (28%)
Colorectal	21 (15%)
Lung	58 (40%)
Prostate	26 (18%)
Class of drugs	
Chemotherapy	38 (26%)
Endocrine therapy	13 (9%)
Targeted agents	85 (59%)
Immunotherapy	9 (6%)
Others	7 (5%)
Sponsorship	
Industry	128 (90%)
Other	15 (10%)
Journal impact factor	
<5	18 (12%)
5‐10	15 (10%)
>10	99 (69%)
HRQOL tool used	
EORTC QLQ	19 (32%)
EuroQol‐5D (EQ‐5D)	12 (20%)
FACT	23 (39%)
Other	11 (19%)
Not specified	20 (24%)

Note

Some clinical trials included more than one cancer site and more than one type of anti‐cancer agent. HRQoL characteristics from studies that measured and reported PRO data.

C30, core 30; EORTC, European Organisation for Research and Treatment of Cancer; FACT, Functional Assessment of Cancer Therapy; N/A, not available; QLQ, Quality of Life Questionnaire.

### 3.2 Health‐related quality of life data reporting

**FIGURE 2:**
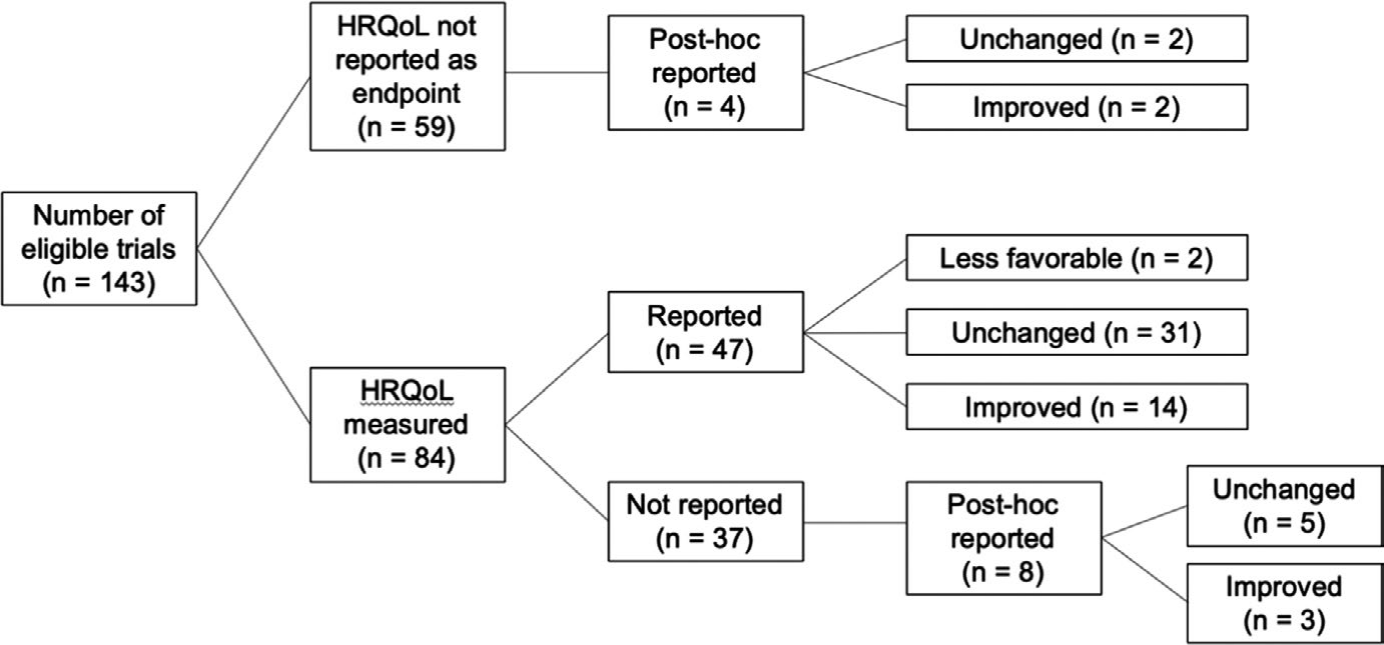
Distribution of HRQoL reporting and outcomes among all eligible clinical trials. HRQoL, Health‐related quality of life

As shown in Figure 2, of the 143 studies in our dataset, 84 (59%) trials reported collecting HRQoL data and among these 47 (56%) reported these data concurrently with the primary analysis. Of these 47 trials, 14 (30%) showed improvement in HRQoL, 2 (4%) showed less favorable HRQoL, and 31 (66%) showed no change. Of the 37 trials that did not report QoL data initially, 8 (22%) reported HRQoL data after the primary trial results were reported and/or published. Among these, 5 (63%) showed no change in HRQoL and 3 (38%) showed improvement. There was no trend for change in the reporting of HRQoL over time (*P* for trend = 0.97).

### 3.3 Association of treatment tolerability and toxicity with HRQoL reporting

The proportion of studies that reported on specific safety and tolerability outcomes is summarized in Table 2. Treatment discontinuation was reported most often (76%) among all variables analysed whereas neuropathy was reported least often (28%). There was no association between reporting of HRQoL data and treatment‐related death or treatment discontinuation. The odds of reporting HRQoL was negatively associated with dyspnea and positively associated with dermatological toxicity. Associations with grade 3 or 4 adverse events (AEs) are summarized in Table 2.

**TABLE 2:** Proportion of death, treatment tolerability, and toxicities reported by all eligible trials and associations of HRQoL reporting

Variable	n (%)	OR	95% CI	*P*‐value
Death	102 (71%)	1.03	(0.60‐1.77)	0.91
Treatment discontinuation	109 (76%)	1.16	(0.74‐1.82)	0.51
Individual adverse events and toxicities				
Dermatological	78 (54%)	1.60	(1.12‐2.27)	0.01
Hypertension	46 (32%)	1.29	(0.63‐2.66)	0.49
Vomiting	81 (57%)	1.25	(0.77‐2.04)	0.37
Cardiotoxicity	45 (31%)	1.15	(0.41‐3.23)	0.79
Anemia	84 (59%)	1.04	(0.70‐1.55)	0.84
Diarrhea	102 (71%)	1.04	(0.77‐1.41)	0.80
Neutropenia	93 (65%)	1.02	(0.79‐1.31)	0.91
Stomatitis	51 (36%)	1.01	(0.61‐1.65)	0.98
Neuropathy	40 (28%)	0.97	(0.64‐1.48)	0.91
Fatigue	104 (73%)	0.94	(0.58‐1.54)	0.82
Thrombocytopenia	43 (30%)	0.75	(0.44‐1.29)	0.29
Dyspnea	53 (37%)	0.20	(0.046‐0.88)	0.04

### 3.4 Association of clinical trial characteristics and HRQoL reporting

Associations between clinical trial characteristics and odds of reporting of HRQoL are summarized in Tables 3 and 4. Positive trials were more than twice as likely to report HRQoL data compared to trials which did not meet their primary endpoints. HRQoL was also more likely when published in journals with higher impact factor. All other characteristics including cancer site and trial sponsorship were not found to be significantly associated with HRQoL reporting. No statistically significant relationship was found between the type of PRO tool used (EORTC QLQ C30, FACT, EQ5D, and other) and HRQoL data reporting (see Table 4.

**TABLE 3:** Association of HRQOL reporting and clinical trial features

Variable	OR	95% CI	*P*‐value
Trial Sponsorship (industry vs other)	2.08	(0.63‐6.86)	0.23
Outcome of trial (positive vs negative)	2.05	(1.03‐4.09)	0.04
Journal Impact Factor (for every 10‐unit change in impact factor)	1.35	(1.10‐1.67)	0.01
Cancer site			
Prostate	Reference		
Breast	1.24	(0.44‐3.49)	0.69
Colon	0.56	(0.16‐2.03)	0.38
Lung	1.72	(0.66‐4.52)	0.28
Other	Not measurable		

**TABLE 4:** HRQoL tool used and reporting relationship

HRQoL tool	Odds ratio	95% CI	*P*‐value
EORTC QLQ C30	1.77	0.58‐5.44	0.58
FACT	1.71	0.65‐4.48	0.28
EQ‐5D	0.70	0.24‐2.07	0.52
Other	0.48	0.18‐1.31	0.16

Abbreviations: EORTC QLQ C30, European Organization for the Research and Treatment of Cancer Quality of Life Questionnaire Cancer 30 questions; EQ5D, EuroQol 5D version; FACT, Functional Assessment of Cancer Therapy.

## 4 DISCUSSION

Information about the impact that anti‐cancer drugs have on the quality of life of cancer patients is essential for both oncologists and patients. HRQoL assessment is a direct measure of patient experience and is an important clinical endpoint when evaluating anti‐cancer drugs. Over the last decade, the availability of HRQoL data has been highlighted as important when counselling patients on particular treatment regimens.^19^ Given the growing demand for improved reporting of PRO data to help patients make informed decisions,^20^, ^21^ efforts have been made to guide investigators on how to plan the collection of high‐quality PRO data in clinical trials^22^ and how to optimize reporting of PRO results.^23^, ^24^ This reflects the increase in patient‐centred evaluation of cancer treatment as well as the need to quantify the impact of cancer interventions from an economic standpoint.^25^, ^26^, ^27^ Despite these efforts and the availability of validated HRQoL assessment tools, the reporting of PRO data in randomized phase III clinical trials remains low. Analyses suggest that only around 50% of phase III cancer trials specify collection of HRQoL data and fewer still report these data.^28^, ^29^

To further investigate this, we explored whether the safety and tolerability profile of anti‐cancer drugs is associated with the odds of reporting of HRQoL. Among the 143 phase III trials, 84 reported the collection of HRQoL data and among these, treatment‐related death, treatment discontinuation, and most AEs were not associated significantly with reporting of HRQoL data. These findings are in contrast to our initial hypothesis and are reassuring for the absence of selective reporting of HRQoL based on the toxicity of cancer drugs. Although various studies have evaluated whether HRQoL is impacted by cancer treatments and have also identified factors that impact patient HRQoL,^30^, ^31^, ^32^ our analysis adds to this body of knowledge and suggests no association between safety and tolerability of individual cancer drugs and HRQoL. Of interest, we did find an association between dyspnea and a lower odds of reporting HRQoL. While the magnitude of this association was relatively large (five‐fold decrease in odds), statistical significance was borderline and may reflect a false discovery. However, it remains possible than dyspnea has a greater impact on HRQoL than other toxicities and that this may explain this observation. The association between dermatological toxicity and higher odds of HRQoL reporting was of low magnitude and more likely reflects a false discovery.

It is important to note that our analysis was based on safety and tolerability outcomes within the corresponding phase III trial in which HRQoL was assessed. The safety and tolerability signals from early phase clinical trials still may have an important role for the design of phase III trials including plans to collect HRQoL data. In our study, HRQoL data collection was planned in only around 60% of trials. The reasons for failure to list HRQoL as an endpoint is uncertain. We cannot rule out that prior knowledge of the safety and tolerability of the experimental drug in earlier phase studies may impact on decisions to collect HRQoL data in phase III trials. Furthermore, in some trials in which HRQoL data collection were planned, these outcomes may not have been collected and consequently not reported.

Of note, in contrast to prior studies,^33^ our findings do not suggest an improvement in the reporting of HRQoL over time. This underscores the inconsistent access to clinically relevant information on the impact of treatment on HRQoL and may result in suboptimal decision making for patients and their healthcare team.^34^

The reasons for the low level of reporting of HRQoL remain unclear. One possible explanation is related to trial outcome. Previous studies have revealed a relationship between HRQoL reporting and trial outcomes.^13^, ^35^ Interestingly, while the proportion of trials reporting PRO data was similar among positive and negative trials when only the initial publication was assessed, among positive trials, the proportion reporting HRQoL increased when including secondary publications.^13^ Our analysis confirms an increased probability of HRQoL reporting with positive trial outcomes. A potential explanation for this is that anti‐cancer drugs that do not show efficacy will not be approved by regulators and therefore additional publication of HRQoL data is not pursued, despite its availability. Another possible explanation is the lack of requirements by regulators to include HRQoL data as an endpoint.

Journal editors could mandate the reporting of HRQoL data if they are listed as endpoints in respective trials. This idea is supported by our findings that HRQoL data are more likely to be reported if the trial is published in a journal with a higher impact factor. It is unclear whether these journals required publication of these findings or if this observation was influenced by positive trials being more likely to be published in higher impact journals. Nonetheless, these findings demonstrate a concerning finding that trials published in high impact journals or those with positive results preferentially report HRQoL data. As reporting of HRQoL is associated with positive trials it is not surprising that among trials which reported HRQoL concurrently with primary efficacy data, results were less favourable in the experimental arm in fewer than 5% of cases. However, in the light of known toxicity of modern targeted and combination therapies^36^ this may indicate a bias in reporting of HRQoL within RCTs. This further reinforces a recommendation that all journals require HRQoL be reported, if collected, to respect the patients enrolled in the trial who took time to provide these data.

Our analysis also revealed that across the four most common cancer types, utilization of specific HRQoL assessment tools varies substantially. For example, although the EORTC quality of life questionnaire core 30 (QLQ‐C30) is the most commonly used in clinical trials,^33^, ^37^, ^38^, ^39^ our study reveals that it was used in only one quarter of the studies that were eligible for analysis. Currently, there is no recommendation for the use of a specific assessment tool. Although the different HRQoL tools used in the included trials are well validated, standardization of one tool may allow more consistent assessment of magnitudes of effect and aid in cross trial comparisons if warranted.

This study has limitations. First, with trials of cancer drugs in less common solid tumors being more commonly single arm than randomized trials,^40^ we elected to focus only on RCTs in four common solid tumors, thereby having a more homogeneous cohort of large phase III trials. However, this trade‐off may have resulted in reduced generalizability. Reports from other, less common tumor sites may have more reliable HRQoL reporting such as been reported in gynecological cancers.^33^ Second, given that we included phase III RCTs completed and reported between 2005 and 2016, we may have also missed changes in HRQoL reporting over subsequent years, especially in the context of increasing patient‐centred focus of drug approval.^25^ However, we aimed to have a number of years of follow‐up after trial completion to allow for HRQoL to be reported. Third, our focus on phase III RCTs will have resulted in us capturing only a proportion of all available safety, tolerability, and HRQoL data. Those from earlier phase I and II trials, which have a greater focus on drug safety and tolerability would not have been included.^41^, ^42^ Fourth, reporting of safety and tolerability was not consistent among trials. This can lead to under‐estimation of the true influence of toxicity on HRQoL reporting. Fifth, as we analysed single adverse events and their association with HRQoL reporting, we did not evaluate whether an interaction between specific AEs specific may impact HRQoL reporting. Finally, assessment of study quality was not feasible in a large number of included trials. In many trials, blinding was deemed ineffective as a result of the potential for unblinding due to differential toxicity (eg alopecia in trials comparing cytotoxic chemotherapy to targeted therapy). While this may not have been crucial for the assessment of the primary endpoints of included trials (which were typically efficacy‐based), it may have impacted our focus on toxicities and HRQoL. With none of the validated study quality tools having a focus on reporting of secondary outcome measures, we did not feel that assessment of study quality would be meaningful.

In conclusion, HRQoL is reported in only two‐thirds of RCTs in common solid cancers that aim to collect such data. Reporting of HRQoL is associated with positive trial outcome and with publication in higher impact journals, but is not associated with the overall safety and tolerability of anti‐cancer drugs. As HRQoL is a direct measure of patient outcomes, trialists should be encouraged to collect such data and efforts should be made to improve the reporting of these important measures.

## CONFLICT OF INTEREST

Dr Eitan Amir reports personal fees from Genentech/Roche, personal fees from Apobiologix, personal fees from Myriad Genetics, personal fees from Agendia, outside the submitted work. Dr Ramy Saleh reports personal fees from Roche, outside the submitted work. Dr Nicholas Meti reports personal fees from Novartis, outside the submitted work. Dr Domen Ribnikar reports personal fees from Novartis, outside the submitted work. Dr Hadar Goldvaser reports personal honorarium fees from Roche, Novartis, Pfizer and Oncotest, personal advisory consultation fees from Novartis, all outside the submitted work. Dr Alberto Ocana reports travel support from Merck, advisory consultation fees from Daichii‐Sankyo and Entrechem. Dr Arnoud J. Templeton reports personal fees from Astellas, MSD, and Sanofi. Consultancy fees were paid to his institution by BMS, Janssen, Sanofi, and Roche. Conference/travel support was received from Sanofi, Janssen, Ipsen, and Roche. Dr Bostjan Seruga reports personal honorarium fees from Astellas, Jansse, Novartis and Roche.

## AUTHOR CONTRIBUTIONS

Eitan Amir and Ramy Saleh contributed to study concept and design. Ramy Saleh and Domen Ribnikar contributed to data acquisition. Ramy Saleh, Eitan Amir, and Nicholas Meti contributed to data analysis. Ramy Saleh, Eitan Amir, and Nicholas Meti contributed to statistical analysis. Ramy Saleh, Eitan Amir, and Nicholas Meti contributed to manuscript preparation. All authors contributed to manuscript editing and review.

## Data Availability

The data that support the findings of this study are available from the corresponding author upon reasonable request.
